# Impact of intense pulsed light irradiation on cultured primary fibroblasts and a vascular endothelial cell line

**DOI:** 10.3892/etm.2012.664

**Published:** 2012-08-14

**Authors:** DI WU, BINGRONG ZHOU, YANG XU, ZHIQIANG YIN, DAN LUO

**Affiliations:** Department of Dermatology, The First Affiliated Hospital, Nanjing Medical University, Nanjing, Jiangsu 210029, P.R. China

**Keywords:** intense pulsed light, collagen, matrix metalloproteinases, vascular endothelial growth factor, fibroblasts

## Abstract

The aim of this study was to determine the effects of intense pulsed light (IPL) on cell proliferation and the secretion of vascular endothelial growth factor (VEGF) and matrix metalloproteinases (MMPs) in human fibroblasts and vascular endothelial cell lines, and to investigate the effects of IPL on the mRNA expression levels of type I and III procollagens in cultured human fibroblasts. Foreskin fibroblasts and a vascular endothelial cell line (ECV034) were cultured and treated with various wavelengths and doses of IPL irradiation. After culture for 1, 12, 24 and 48 h following IPL irradiation, fibroblasts and the vascular endothelial cell line were harvested for investigation of morphological changes by light microscopy, cell proliferation viability by MTT assay, and VEGF and MMP secretions by ELISA. The mRNA expression levels of type I and III procollagens in the fibroblasts were detected by RT-PCR. No marked morphological changes were observed in the cultured fibroblasts compared with the control. Cell growth and cellular viability were increased in fibroblasts 24 and 48 h after IPL irradiation. The levels of type I and III procollagen mRNA expression in fibroblasts increased in a time-dependent manner. However, the IPL management had no impact on VEGF and MMP secretion levels in fibroblasts and the ECV034 cell line at any time-point after irradiation as well as cell morphology and cellular proliferation. IPL irradiation may induce cellular proliferation and promote the expression of procollagen mRNAs directly in cultured primary fibroblasts, which may primarily contribute to photorejuvenation.

## Introduction

The aging of human skin includes intrinsic aging and photoaging, characterized by a thining epidermis, decrease in collagens and deposition of abnormal elastic fibers (1). It is well-documented that human fibroblasts generate matrix metalloproteinases (MMPs), which specifically degrade the majority of the extracellular matrix (ECM) components and play an important role in skin natural aging and photoaging (2). Vascular endothelial growth factor (VEGF) is the only growth factor with a specific effect on angiogenesis. After stimulation, vascular endothelial cells and other cells produce VEGF, which may be involved in the process of inflammation and carcinogenesis (3).

The photorejuvenation technique is a type of non-ablative treatment using continuous wavelength intense pulsed light (IPL) at low energy density (4). Photorejuvenation is mostly used to treat photoaging and telangiectasias, among others (5). The underlying mechanism is associated with the photothermal and photochemical effects generated by IPL (6).

To date, no data are clear on the effects of IPL on human skin cells and the associated mechanism. Our research aimed to investigate the effects of IPL on cell proliferation and protein secretion of VEGF and/or MMP-1 and MMP-2 in human fibroblasts and vascular endothelial cells, and to study the effects of IPL on mRNA expression levels of procollagen type I and III in cultured human fibroblasts.

## Materials and methods

### Cell culture and IPL irradiation

Fibroblasts were isolated from circumcised foreskin (using 0.5% dispase, 0.25% trypsinase and 0.1% EDTA). Fibroblasts and a vascular endothelial cell line (ECV034) were cultured in medium and plated in 35-mm dishes and/or 96-well plates with the same amounts of cells. Subconfluent primary fibroblasts and the vascular endothelial cell line were irradiated with IPL with a certain wavelength and dosage (wavelength 520–1200 nm, 5 pulses, pulse width 15 msec, pulse delay 5 msec, fluence 15, 29 and 35 mJ/cm^2^). After 24-h culture, cells were collected for morphological observation by light microscopy and the following procedures.

### Cell viability assay

Cellular viability was measured by MTT [3-(4, 5-dimethyl-thiazol-2-yl)-2, 5-diphenyldiphenyltetrazoliumbromide] assay. A 5 mg/ml MTT solution (20 μl) was added per 100 μl medium. After 4 h, 100 μl of dimethylsulfoxide (DMSO) was added to each well and the absorbance (A) values at 490 nm were recorded on the microplate reader.

### Detection of certain cytokines

After IPL irradiation, the conditioned medium was collected at various time intervals for ELISA. VEGF, MMP-1 and MMP-2 were detected with the Human Cytokine Sandwich ELISA kit (JingMei Bioengineer Company, Shenzhen, China). According to the instructions, 50 μl samples, specifically diluted antibody, enzyme reagent and color reagent were added and washed step by step in the pre-coated 96-well immunoplates. The standard curve demonstrated a direct association between OD values and secreted cytokine levels.

### Detection of mRNA expression of type I and III procollagens

The total mRNA of fibroblasts was harvested by Tri-reagent (Gibco-BRL, Gaithersburg, MD, USA) after IPL irradiation. The ratio of OD260/OD280 was between 1.8–2.0, suggesting no degradation and no protein contamination. DNA was denatured at 94°C for 6 sec and PCR was performed immediately for 30 cycles, with denaturation at 94°C for 50 sec, annealing at 55°C for 50 sec, extension at 72°C for 70 sec and the final extension at 72°C for 7 min. Table I lists the primer sequences and the expected length of type I and III procollagens in RT-PCR amplification. The RT-PCR products were run through gel electrophoresis using 2% agarose gel. The quantitative analysis of the optical density of the cDNA bands was performed by Bio-Rad Quantity One^®^ Image software. The density value of each band of collagen type I and III was divided by the density value of its corresponding GAPDH band for normalization. The resultant values were expressed as relative intensities.

### Statistical analysis

The experimental data are expressed as mean ± SD and analyzed by SPSS 10.0 software. P<0.05 was considered to indicate a statistically significant result.

## Results

### Effect of IPL irradiation on the morphology of skin fibroblasts

**Figure 1:**
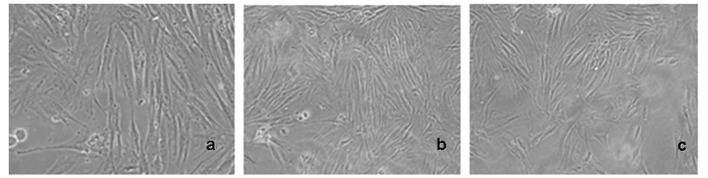
Morphology of fibroblasts 24 h after IPL irradiation. Primary fibroblasts isolated from the foreskin were cultured in 10% CSF medium following various dosages of IPL irradiation. Morphological changes could be observed under the microscope after 24 h of culture. (a) Change with sham irradiation; (b) results following IPL irradiation at 18 and (c) 35 J/cm^2^. IPL, intense pulsed light. Magnification, x40.

Subconfluent primary fibroblasts were treated with doses of IPL irradiation (18, 29 and 35 J/cm^2^, wavelength 590–1,200 nm) and cultured for 24 h. There was no clear morphological change observed compared with the non-irradiated fibroblasts under light microscope by the end of the experiment (Fig. 1).

### Effect of IPL on the cell proliferation of skin fibroblasts

**Figure 2:**
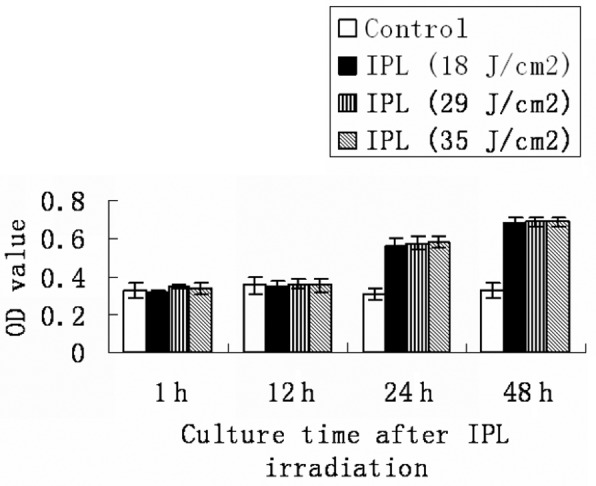
Effects of IPL irradiation on fibroblast proliferation. Primary fibroblasts isolated from foreskin were cultured from 1 to 48 h in 10% CSF medium after various dosages of IPL irradiation. MTT assay was used to detect cellular viability. The time effect of IPL irradiation on cellular viability is shown, which had no notable effect within 24 h of culture, but there was a statistical difference between each dose of IPL irradiation and sham control after culturing the fibroblasts from 24 to 48 h. IPL, intense pulsed light.

Fibroblasts were treated with doses of IPL irradiation (18, 29 and 35 J/cm^2^). Fig. 2 and Table II show that there is no significant difference in fibroblast viability or proliferation at the time-points of 1 or 12 h after IPL irradiation (P>0.05). However, the cellular viability and proliferation of fibroblasts increased clearly after 24- and 48-h IPL treatment compared with the controls (P<0.05).

### Effect of IPL irradiation on cell proliferation of vascular endothelial cell line

**Figure 3:**
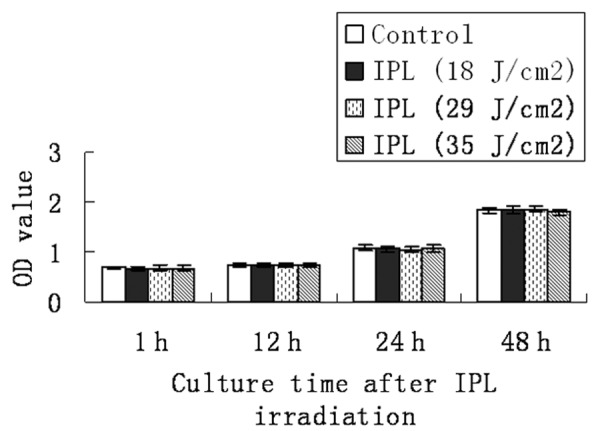
Effect of IPL irradiation on cell proliferation of the vascular endothelial cell line. The vascular endothelial cell line was cultured in 10% CSF medium after various dosages of IPL irradiation and cultured from 1 to 48 h. MTT assay was used to detect cellular viability. The time effect of IPL irradiation on cellular viability is shown, with no notable effect within 48 h of culture between each dose group of IPL irradiation and sham control. IPL, intense pulsed light.

Subconfluent vascular endothelial cell line (ECV034) was treated with the same doses of IPL irradiation and culture time as mentioned above. No obvious changes in cellular viability and proliferation in the vascular endothelial cell line were observed by the end of our experiment (P>0.05; Fig. 3 and Table III).

### Effect of IPL on VEGF secretion in primary fibroblasts and vascular endothelial cells

**Figure 4:**
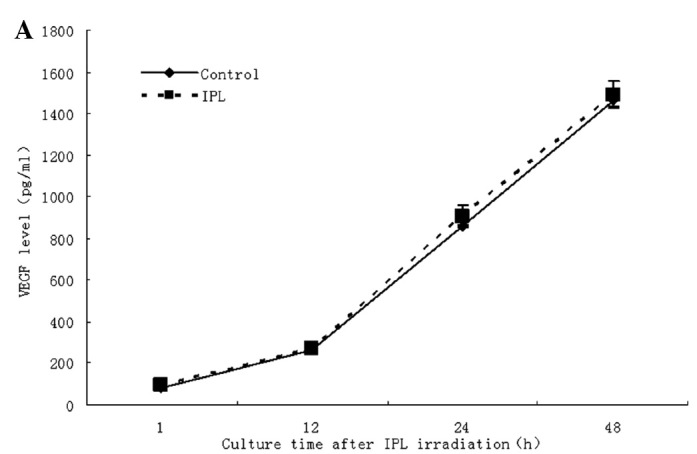

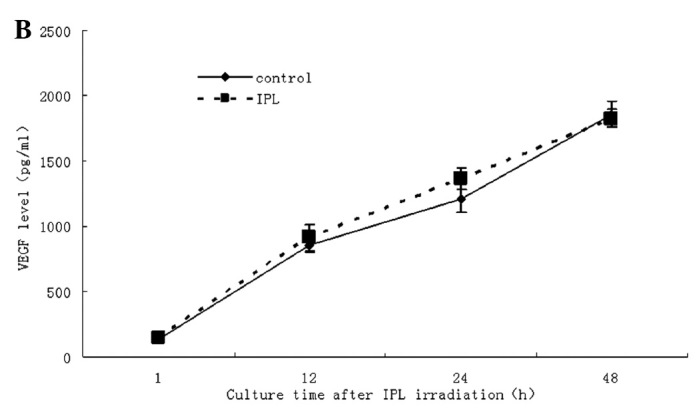
Time effect of IPL irradiation on VEGF secretion from fibroblasts and the vascular endothelial cell line. To investigate whether IPL had direct or indirect effects on fibroblasts and ECV034 vascular endothelial cells, they were treated with various wavelengths and doses of IPL irradiation (590–1,200 nm; 18, 29 and 36 J/cm^2^). By culturing for 1, 12, 24 and 48 h after the irradiation, the secreted proteins of VEGF from cell supernatants were determined by ELISA. No obvious regulatory change in VEGF secretion was observed in fibroblasts or the ECV034 cell line.

To investigate the effects of IPL, fibroblasts and the vascular endothelial cell line (ECV034) were treated with various wavelengths and doses of IPL irradiation (590–1200 nm and 18, 29 and 35 J/cm^2^). After culturing for 1, 12, 24 and 48 h after irradiation, the levels of VEGF in cell supernatants were determined by ELISA. No clear regulation of VEGF secretion was observed in fibroblasts and the ECV034 cell line (Fig. 4A and B).

### Effect of IPL on mRNA expression of procollagen type I and III in fibroblasts

**Figure 5:**
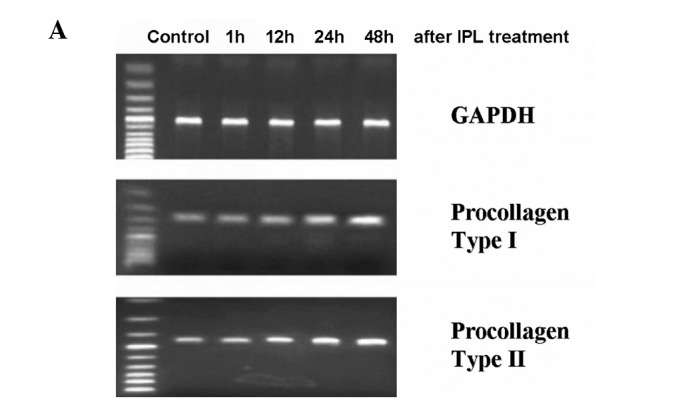

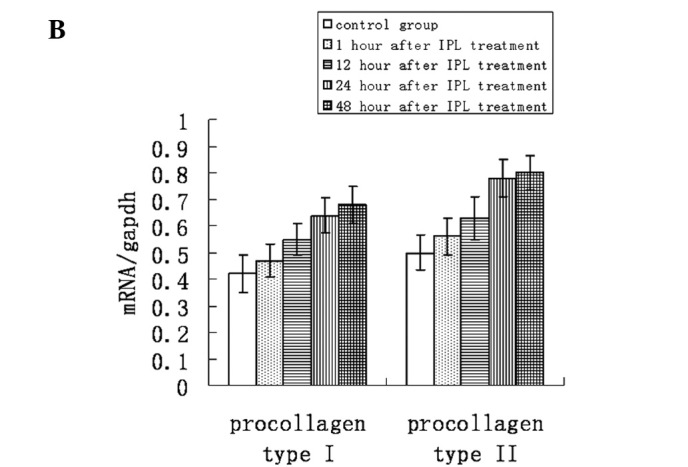
Effect of IPL irradiation on mRNA expression of type I and III procollagens of cultured skin fibroblasts. Cultured fibroblasts were treated with IPL irradiation (570–590 nm and 18 J/cm^2^) and the mRNA expression was measured after culturing irradiated fibroblasts for 1, 12, 24 and 48 h. Compared with sham irradiation, the mRNA expression levels of procollagen type I and III did not increase noticeably after cultured from 1 to 12 h. After the irradiated fibroblasts were cultured for 24 and 48 h, the expression levels of the procollagens were obviously increased (P<0.05) and the upregulatory effect of IPL was also time-dependent.

The cultured fibroblasts were treated with IPL irradiation (570–590 nm and 18 J/cm^2^) and the mRNA expression was measured after culturing irradiated fibroblasts for 1, 12, 24 and 48 h. Compared with sham irradiation, the mRNA expression levels of procollagen types I and III did not increase markedly after short-term culture. The procollagen levels were clearly increased (P<0.05) when the irradiated fibroblasts were cultured from 24 to 48 h after IPL irradiation and the upregulation effect of IPL was also time-dependent (Fig. 5A and B).

### Effect of IPL irradiation on the secretion of MMP-1 and MMP-2 in fibroblasts

The fibroblasts were irradiated by IPL (590–1200 nm, 29 J/cm^2^) and cultured for 24 h. The conditioned supernatant was collected and measured by ELISA for levels of MMP-1 and MMP-2. The results showed that IPL irradiation had no significant effect on the secretion levels of MMP-1 and MMP-2 proteins (P>0.05; Table IV).

## Discussion

The development of non-ablative laser and light treatment provide an alternative to the traditional ablative modalities and improve overall skin texture and tone. Non-ablative laser and light therapy (also termed photorejuvenation) is a relatively new concept for the impovement of the visual appearance of photodamaged skin and acne scars. The remarkable advantage of non-ablative therapy is the limited downtime after treatment.

IPL is a laser-like device that uses a flash lamp to produce a non-coherent pulsed light from 515 to 1,200 nm with variable pulse durations and intervals. Photorejuvenation is mainly used in the treatment of certain skin diseases, including photoaging (7) and telangiectasias (8). IPL also works on dermal cells, fibers and vessels (9) by the principle of selective photothermolysis. The major advantage of this device is its versatility in providing the wide range of wavelengths, pulse durations and pulse delay that allow the treatment of numerous types of lesions. This versatility makes IPL able to adjust to the type, depth and the size of the lesion as well as the skin type of the patient to achieve maximum clearance (10). The advantages of the new IPL technique are manifold, with optimal treatment parameters, including pulse and wave-OPT system, and almost without epidermal loss, side effects or recovery periods.

It is well-known that collagen in the dermis is mainly composed of type I (80%) and III (10%) collagens, which are responsible for the elasticity and integrity of the skin. Fibroblasts produce and secrete procollagen which consists of collagens. For non-ablative therapy, an epidermal surface temperature of 40–48°C, is ideal since this correlates with a dermal temperature of 55–65°C, which is required for collagen denaturation. It was reported by Talwar *et al* (11) that procollagen type I and III levels decreased in photoaging and/or aged skin. After IPL treatment, fibroblasts promote the production of procollagen, which may have some association with IPL by direct stimulation and/or photothermolysis. In this way IPL increases the substantial production and rearrangement of collagens in the dermis (4,6) and makes the skin more elastic and rejuvenated.

Our research focused on primary fibroblasts and the vascular endothelial cell line (ECV034) to confirm whether IPL irradiation could increase the production of procollagen from cultured fibroblasts and/or increase endothelial cell proliferation and VEGF production. There was no morphological change observed under light microscopy, which suggests that IPL has no heat-injury effect on cultured fibroblasts (Fig. 1). In addition, the cell proliferation of fibroblasts increased dramatically 24 and 48 h after administration of a series of doses (18, 29 and 35 J/cm^2^) of IPL irradiation compared with sham irradiation but IPL showed no effect on the ECV034 cell line (Tables II and III, Figs. 2 and 3). The RT-PCR assay also revealed mRNA expression levels of the procollagen type I and III from fibroblasts were increased at 12, 24 and 48 h after IPL irradiation (Fig. 5). ELISA results revealed that IPL could stimulate fibroblasts to secret type I and III procollagens and consequently increase the level of collagens. Our study demonstrated that IPL irradiation on Chinese Han fibroblasts resulted in increased cell proliferation and procollagen mRNA levels.

VEGF is the most important cytokine of the vascular endothelial growth factor family, which plays an important role in the growth and differentiation of vascular, as well as lymphatic, endothelial cells. VEGF is the pivotal angiogenic growth factor activating endothelial cells to migrate, proliferate and form capillary tubes (12). VEGF induces increased vascular permeability, angiogenesis and then the skin repair process. In this study, IPL showed no up- or downregulatory effects on VEGF levels in fibroblasts or vascular endothelial cells at any time-point after IPL irradiation (Fig. 4). The data showed that IPL has no direct effects on the cellular proliferation or cytokine secretion from the vascular endothelial cell line, which supports that the mechanism of IPL on telangiectasias has no association with VEGF. It may have an association with the destruction and deposition of vascular endothelial cells after IPL photothermolysis.

With regard to human skin aging, it is not only associated with the decrease in new collagen production, but also with the increase of collagen degradation. MMPs are a group of ECM enzymes that degrade all known protein components of the ECM (13). The upregulation of MMPs, particularly collagenase-1 (MMP-1), stromelysin-1 (MMP-3) and gelatinase A (MMP-2), is responsible for the lysis of dermal collagen and elastic fibers during skin aging (14). MMPs originating from keratinocytes and fibroblasts are considered to play a primary role in this process. In response to UV irradiation, mitogen-activated protein kinase signaling pathways are activated mediating the upregulation of MMP expression. However, in response to IPL irradiation, there was no upregulation of the secretion levels of MMP-1 and MMP-2 proteins on fibroblasts (Table IV), which suggests that IPL irradiation could not degrade the protein components of ECM as does UV irradiation.

IPL irradiation induced cell proliferation in cultured primary fibroblasts but had no such effect on the vascular endothelial cell line. IPL had no regulatory effect on VEGF secretion for either cultured fibroblasts or ECV304. In addition, IPL had no upregulatory effect on MMP-1 and MMP-2 secretion from fibroblasts. However, IPL irradiation promoted the transcription of type I and III procollagen mRNA in fibroblasts directly, which suggests at a part of the mechanism of photorejuvenation.

## Figures and Tables

**Table I:** Primers used in the RT-PCR amplification of the human procollagen type I and III and GAPDH mRNAs.

Primer	Sequence	Length of sequence (bp)
Collagen I		
Upstream	5′-CTGGTCCCAAGGGTAACAG-3′	285
Downstream	5′-GCCAGGAGAACCACGTTC-3′	
Collagen III		
Upstream	5′-CTGCCATCCTGAACTCAAGAGTGG-3′	447
Downstream	5′-CCATCCTCCAGAACTGTGTAGG-3′	
GAPDH (control)		
Upstream	5′-AACCATGAGAAGTATGACAACAGC-3′	580
Downstream	5′-CATGTGGGGCCATGAGGTCCACCAC-3′	

bp, number of base pairs.

**Table II:** Time effects of IPL on the cell proliferation of primary fibroblasts.

	Culture time after IPL radiation (h)			
Treatment	1	12	24	48
Sham	0.329±0.038	0.352±0.042	0.307±0.028	0.329±0.038
IPL (18 J/cm^2^)	0.313±0.012	0.344±0.036	0.565±0.040^a^	0.678±0.032^b^
IPL (29 J/cm^2^)	0.344±0.012	0.358±0.023	0.577±0.038^a^	0.688±0.027^b^
IPL (35 J/cm^2^)	0.336±0.028	0.351±0.031	0.582±0.029^a^	0.688±0.027^b^

a P<0.05 compared with sham goup;

b P<0.01 compared with sham goup. IPL, intense pulsed light. Data are the absorbance values (mean ± SD).

**Table III:** Time effects of IPL irradiation on cell proliferation of vascular endothelial cell line.

	Culture time after IPL radiation (h)			
Treatment	1	12	24	48
Sham	0.689±0.030	0.739±0.052	1.086±0.059	1.847±0.056
IPL (18 J/cm^2^)	0.671±0.040	0.725±0.035	1.066±0.062	1.856±0.072
IPL (29 J/cm^2^)	0.674±0.050	0.723±0.037	1.051±0.059	1.868±0.061
IPL (35 J/cm^2^)	0.674±0.050	0.738±0.040	1.079±0.061	1.802±0.063

IPL, intense pulsed light. Data are the absorbance values (mean ± SD).

**Table IV:** Effects of IPL irradiation on MMP-1 and MMP-2 secretion from fibroblasts.

MMP (ng/ml)	Controls	IPL (29 J/cm^2^)
MMP-1	3.89±0.16	3.75±0.18
MMP-2	1.50±0.14	1.41±0.11

IPL, intense pulsed light; MMP, matrix metalloproteinases. Data are the absorbance values (mean ± SD).
